# Intertwin nuchal translucency difference predicts the chance of co-twin death after fetal reduction in dichorionic triplet pregnancies: a retrospective analysis study

**DOI:** 10.1186/s12884-023-06064-9

**Published:** 2023-10-23

**Authors:** Shuting Xia, Kaniok You, Minhuan Lin, Linhuan Huang, Zhiming He, Xuan Huang, Yanmin Luo

**Affiliations:** 1 Department of Obstetrics & Gynecology, The First Affiliated Hospital of Sun Yat-Sen University, No.58, Zhong Shan Er Road, Guangzhou, 510080 People’s Republic of China; 2 Guangdong Provincial Clinical Research Center for Obstetrical and Gynecological Diseases, Guangzhou, People’s Republic of China

**Keywords:** Intertwin nuchal translucency difference, Dichorionic triplets, Multifetal pregnancy reduction, Co-twin death, Twin-to-twin transfusion syndrome

## Abstract

**Objectives:**

To compare the clinical outcomes of different multifetal pregnancy reduction (MFPR) programs in dichorionic (DC) triplets, and explore the association between early ultrasound characteristics and co-twin death after potassium chloride (KCl) injection into one monochorionic (MC) twin.

**Methods:**

We retrospectively reviewed the data of DC triplets who underwent MFPR at our center during 2012–2021. Patients were grouped as follows: intracardiac KCl injection into one MC twin (group A), intracardiac KCl injection into both MC twins simultaneously (group B), and reduction of the singleton fetus (group C) and pregnancy outcomes were compared. Logistic regression was used to determine whether ultrasound measurements at 11–13^+6^ weeks predicted co-twin death and the receiver operator characteristic (ROC) analysis was conducted to assess the predictive performance.

**Results:**

Finally, we enrolled 184 patients. 153 cases were in group A, and 18, 13 cases were in group B and C respectively. Gestational age at the time of MFPR did not differ among the 3 groups (median: $12^\frac67$ weeks). The survival rate was 89.6%, 88.9%, and 92.3% in group A, B, and C respectively, which was comparable among groups. Preterm birth was more common in group C (10/12, 83.3%). After KCl injection into one MC twin, co-twin death occurred in 86.3% cases (132/153) within 1 day; however, 3 patients had 2 live births each, with normal postnatal development. Intertwin nuchal translucency (NT) difference/discordance significantly predicted co-twin death within 1 day after MFPR, and the areas under the ROC curve were 0.694 and 0.689, respectively.

**Conclusions:**

For MFPR in DC triplet pregnancies, reduction of the MC twins results in less preterm birth, and women with KCl injection into either one or both MC twins had similar outcomes. Large intertwin NT difference/discordance was associated with co-twin death within 1 day after KCl injection into one of the MC twins.

**Supplementary Information:**

The online version contains supplementary material available at 10.1186/s12884-023-06064-9.

## Introduction

With the advent of assisted reproductive technology (ART), the incidence of multiple pregnancies has increased, and triplet pregnancies now account for approximately 0.9% of all pregnancies [1, 2]. The incidence rates of neonatal mortality and maternal complications are 8 and 3 times higher, respectively, in triplet pregnancies than in singleton pregnancies [3, 4]. Dichorionic (DC) triplet pregnancies consist of monochorionic (MC) twins and a third fetus with its separate placenta. Moreover, DC triplet pregnancies carry a high risk of complications due to communicating vessels within the placenta, and these complications are similar to those observed in MC twin pregnancies, such as twin-to-twin transfusion syndrome (TTTS) and twin anemia-polycythemia sequence (TAPS) [5, 6]. Therefore, multifetal pregnancy reduction (MFPR) can be provided for parents with DC triplet pregnancies [7], and it is reported that MFPR results in a lower rate of perinatal complications than expectant management [8–10].

Several methods are available for performing MFPR in DC triplet pregnancies, such as radiofrequency ablation, cord occlusion and intracardiac potassium chloride injection [11]. Since the application of radiofrequency ablation or cord occlusion depends on the expertise of clinicians and the availability of equipment, potassium chloride injection is more commonly used in China. It is considered that after potassium chloride injection into one of the MC twins, death of the co-twin occurs due to potassium chloride passage through intertwin vascular anastomoses; however, the co-twin was alive after that in some cases, and the associated factors were still unclear [12, 13]. Studies on MC twin pregnancies have indicated that the ultrasound characteristics in early pregnancy, such as crown-rump length (CRL), nuchal translucency (NT), and amniotic fluid volume (AFV), are related to adverse outcomes like TTTS and single intrauterine fetal death (sIUFD) [14–17], but whether they are associated with co-twin death in MFPR is under investigated.

Consequently, we aimed to (i) compare the clinical outcomes of different MFPR programs in DC triplet pregnancies, including potassium chloride injection into one of the MC twins, and (ii) identify the early ultrasound characteristics associated with co-twin death after potassium chloride injection into one of the MC twins. We hope that our findings will provide insights into the consultation of MFPR programs and the possible signs of vascular anastomoses in MC placentas in early pregnancy.

## Materials and methods

### Study subjects

This study was a retrospective analysis of patients with DC triplet pregnancies who underwent MFPR at the Fetal Medicine Center, First Affiliated Hospital, Sun Yat-sen University (Guangzhou, Guangdong, China), between January 2012 and December 2021. Ethical approval for the study protocol was obtained from the ethics committee of First Affiliated Hospital, Sun Yat-sen University.

In the women who were included in this study, 3 live fetuses and chorionicity were confirmed by ultrasound examination. Chorionicity was evaluated in the first trimester by experienced radiologists, on the basis of the following ultrasound findings: the lambda or T sign in a single placenta, number of placental sites, and evaluation of inter-triplet membranes [18]. The exclusion criteria were conjoined twins, twin reversed arterial perfusion sequence, and incomplete medical records.

### MFPR procedure

All patients underwent counselling during which the potential risks of a triplet pregnancy, and the risks and benefits of MFPR were explained to them in detail. Each participant provided written informed consent to undergo the MFPR procedure. The indications for MFPR were triplets without abnormalities or abnormality in 1 or more fetuses, including severe anatomical abnormality on ultrasound examination, NT thickening, and TTTS. Fetal accessibility and the ultrasound findings informed the selection of the fetus to be reduced. Specifically, the fetus with structural abnormalities and/or abnormal NT on ultrasound examination was reduced. If no fetal abnormalities were detected on the ultrasound examination, 1 of the following 3 programs was selected depending on the patient’s preference and fetal accessibility: potassium chloride injection into one of the MC twins or simultaneously into both MC twins or into the singleton fetus. Fetal reduction was performed by the ultrasound-guided, transabdominal, intracardiac injection of potassium chloride (10% KCl, 1–2 mL for 11–13^+6^ weeks and 3–4 mL for 14–19^+6^ weeks) through a 15-cm-long, 22-G biopsy needle, and additional potassium chloride was injected again if the fetal heartbeat did not disappear. Patients were prescribed prophylactic antibiotics. All MFPR procedures were conducted by experienced doctors at our center. A subsequent visit with an ultrasound examination was required on the day after MFPR. If the fetal heartbeat of the co-twin was normal, patients were informed that the risk of complications such as abortion and premature rupture of membranes caused by a secondary fetal reduction is similar to the risk associated with the first reduction procedure, and the risk of neurodevelopmental disorders is similar to that associated with sIUFD during expectant management. Based on the consultations and parents’ willingness, they made a decision about either undergoing expectant management or a secondary fetal reduction of the co-twin, which was scheduled and performed within 1 week. All patients were followed up with routine prenatal visits after MFPR.

### Data collection

The following data were retrospectively collected: pregnancy characteristics, procedure-related characteristics, pregnancy outcomes, and ultrasound measurements at 11–13^+6^ weeks. The pregnancy characteristics recorded in this study included maternal age, parity, mode of conception (spontaneous *vs*. via ART), indications for MFPR, and gestational age (GA) at the time of MFPR. The procedure-related characteristics included number of fetal punctures and potassium chloride dosage during MFPR. The pregnancy outcomes included pregnancy loss (which was defined as miscarriage or termination of pregnancy before 28 weeks due to post‐MFPR fetal complications such as intrauterine infection and preterm premature rupture of membranes), live birth, neonatal death, GA at delivery, caesarean section, birth weight, low birth weight (LBW; ≥ 1500 to < 2500 g), and very low birth weight (VLBW; < 1500 g). To explore factors that were potentially associated with the death of the MC co-twin after MFPR, we reviewed the ultrasound measurements at 11–13^+6^ weeks, including the intertwin differences in CRL, NT, and AFV (i.e., NT_large_—NT_small_); their discordance $(\mathrm{i}.\mathrm{e}., \frac{{\mathrm{NT}}_{\mathrm{large}}-{\mathrm{NT}}_{\mathrm{small}}}{{\mathrm{NT}}_{\mathrm{large}}}\times 100\%)$; and whether the NT in one or more fetuses was ≥ 95^th^ or ≥ 99^th^ percentile of the reference range for the given CRL. 

### Statistical analysis

Categorical variables were expressed as absolute numbers and percentages, and continuous variables were expressed as medians and interquartile ranges. Outcome measures were compared between groups by using the Fisher exact test in the case of categorical variables and the Mann–Whitney *U*-test in the case of continuous variables. Significance was assumed at 5%, and the post-hoc Bonferroni correction was applied to adjust for multiple comparisons, when required.

Univariable logistic regression analysis was performed to examine the association between the ultrasound measurements at 11–13^+6^ weeks and the risk of the death of MC co-twin after MFPR. Further, multivariable logistic regression analysis was used to determine whether they independently predicted the risk of the death of the MC co-twin after MFPR, with adjustments for pregnancy and maternal characteristics, including maternal age, parity, mode of conception, indication for MFPR, and GA at the time of MFPR. The results were expressed as odds ratios (ORs) with 95% confidence intervals (CIs). To evaluate the predictive value of the models, we constructed receiver operator characteristic (ROC) curves, calculated the areas under the curve (AUCs), and compared them using the pairwise chi-square statistic. All statistical analyses were performed using SPSS for Windows, (*v*26.0, IBM Corp., Armonk, NY, USA; 2019).

## Results

**Fig. 1:**
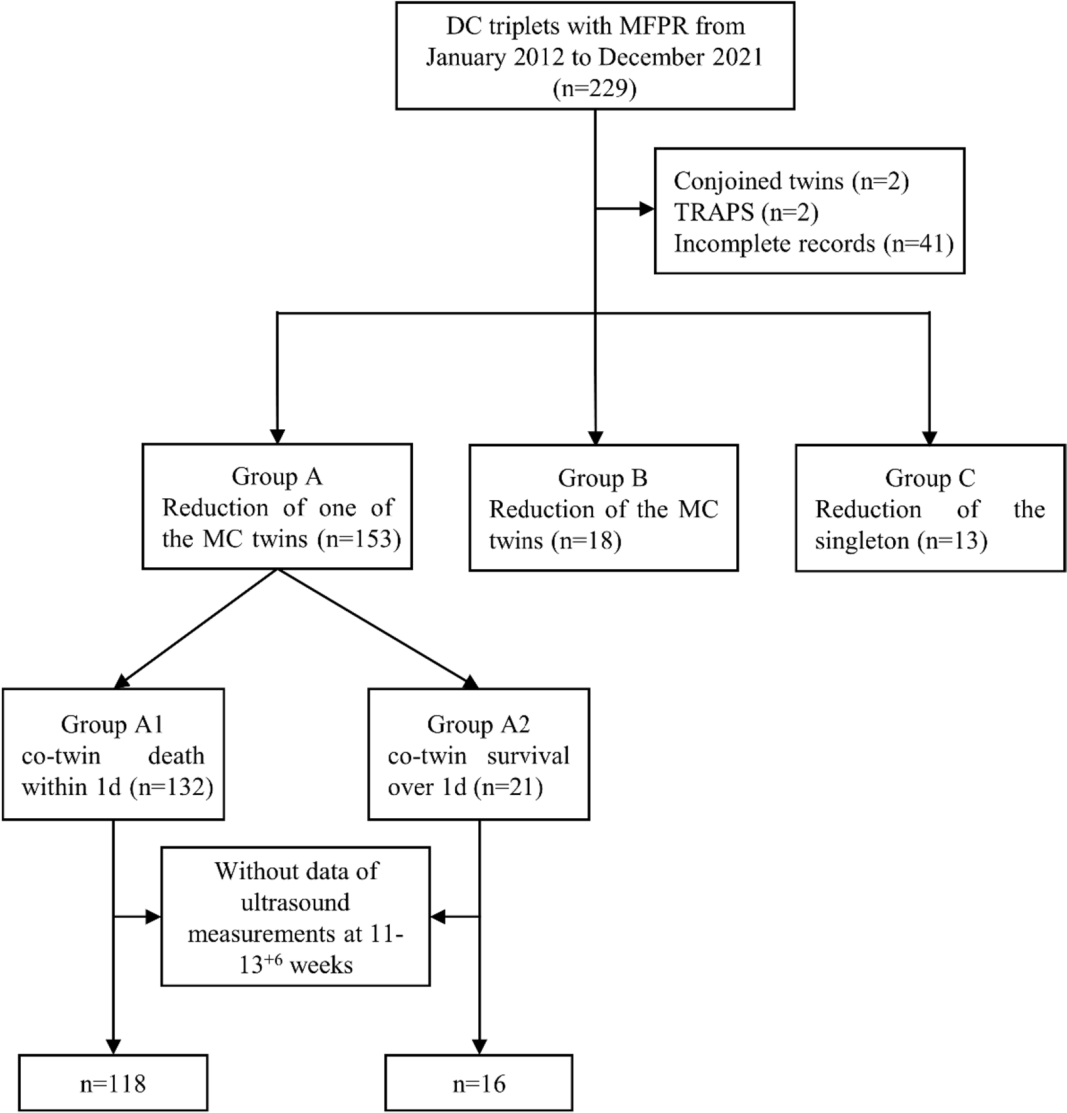
Flowchart summarizing the study design and cases included in the 3 groups. DC, dichorionic, MFPR, multifetal pregnancy reduction, TRAPS, twin reversed arterial perfusion sequence, MC, monochorionic

In total, 229 patients with DC triplet pregnancies underwent MFPR at our center during the study period. Patients with conjoined twins (*n* = 2), twin reversed arterial perfusion sequence (*n* = 2), or incomplete records (*n* = 41) were excluded. Thus, 184 patients with DC triplet pregnancies were included in the present study (Fig. 1). In total, 153 patients opted for intracardiac potassium chloride injection into one of the MC twins (group A), following which, the death of the co-twin occurred within 1 day in 132 cases (group A1), or the co-twin survived for over 1 day in 21 cases (group A2). Among the remaining 31 patients, 18 patients opted for intracardiac potassium chloride injection into both MC twins at the same time to avoid the risk of a secondary fetal reduction (group B), and 13 patients chose to reduce the singleton fetus and continue the pregnancy with the MC twin pair (group C). Pregnancy characteristics, including maternal age, parity, mode of conception, indication for MFPR, and GA at the time of MFPR, did not differ among the group A, B and C (Table 1).

**Table 1:** Comparison of pregnancy characteristics among the 3 groups

**Characteristic**	**Group A** **(*n* = 153)**	**Group B** **(*n* = 18)**	**Group C** **(*n* = 13)**	***P*** **value**
Age (years)	31 (28–34)	32 (28–34)	32 (29–35)	0.864
Parity				0.339
Nulliparous	94 (61.4)	13 (72.2)	6 (46.2)	
Parous	59 (38.6)	5 (27.8)	7 (53.8)	
Mode of conception				0.208
Spontaneous	22 (14.4)	1 (5.6)	0	
ART	131 (85.6)	17 (94.4)	13 (100)	
Indication for MFPR				0.811
Triplets	126 (82.4)	14 (77.8)	10 (76.9)	
Fetal anomaly	27 (17.6)	4 (22.2)	3 (23.1)	
GA at MFPR (weeks)	${12}^{6/7}({12}^{1/7}-{13}^{5/7})$	${13}^{1/7}({12}^{1/7}-{14}^{1/7})$	$13({12}^{1/7}-{13}^{3/7})$	0.630

Data are expressed as median (interquartile range) or n (%)

*ART* Assisted reproduction technology, *MFPR* Multifetal pregnancy reduction, *GA* Gestational age

The pregnancy outcomes of our study population are shown in Table 2. The rate of pregnancy loss was 10.5% (16/153), 11.2% (2/18), and 7.7% (1/13) in groups A, B, and C, respectively; this rate did not significantly differ among the groups. The rates of at least 1 live birth were also similar among the groups. One neonatal death occurred in group A: a baby born at 28 weeks died after the resuscitation effort failed. The median GA at delivery was 39.0 weeks (IQR, 37.8–39.9 weeks), and 39.1 weeks (IQR, 38.3–39.6 weeks) in groups A and B, respectively; in group C, deliveries occurred significantly earlier, at a median GA of 35.8 weeks (IQR, 33.9–36.8 weeks, *P* < 0.001 *vs*. group A or group B). All patients in group C underwent caesarean section (*vs.* 56.2% in group A, *P* < 0.05). Birth weight was significantly lower in group C than in group A or group B (*P* < 0.001), and more than half of the babies in group C had LBW or VLBW.

**Table 2:** Obstetric outcomes among 3 groups

**Outcome**	**Group A** **(*n* = 153)**	**Group B** **(*n* = 18)**	**Group C** **(*n* = 13)**	***P1*** **value**	***P2*** **value**	***P3*** **value**
Pregnancy loss						
Miscarriage	9 (5.9)	1 (5.6)	0	NS	NS	NS
TOP	7 (4.6)	1 (5.6)	1 (7.7)	NS	NS	NS
Live birth						
1	134 (87.6)	16 (88.9)	0	NS	< 0.05	< 0.05
2	3 (2.0)	0	12 (92.3)	NS	< 0.05	< 0.05
At least 1	137 (89.6)	16 (88.9)	12 (92.3)	NS	NS	NS
NND	1 (0.7)	0	0	NS	NS	NS
Delivery						
GA at delivery (weeks)	39.0 (37.8–39.9)	39.1 (38.3–39.6)	35.8 (33.9–36.8)	NS	< 0.001	< 0.001
28–33^+6^ weeks	10 (7.3)	0	3 (25.0)	NS	NS	NS
34–36^+6^ weeks	17 (12.4)	1 (6.3)	7 (58.3)	NS	< 0.05	< 0.05
> 37 weeks	110 (80.3)	15 (93.8)	2 (16.7)	NS	< 0.05	< 0.05
Caesarean section	77 (56.2)	12 (75.0)	12 (100)	NS	< 0.05	NS
Birth weight (g)	3000 (2750–3300)	2975 (2650–3437)	2250 (1985–2500)	NS	< 0.001	< 0.001
≥ 2500 g	116 (84.7)	14 (87.5)	4 (33.3)	NS	< 0.05	< 0.05
LBW	18 (13.1)	2 (12.5)	6 (50.0)	NS	< 0.05	NS
VLBW	3 (2.2)	0	2 (16.7)	NS	< 0.05	NS

Data are expressed as median (interquartile range) or n (%)

*P1*, group A *vs*. group B; *P2*, group A *vs*. group C; *P3*, group B *vs*. group C

*NS* Non-significant (*P* < 0.05), *TOP* Termination of pregnancy, *NND* Neonatal death, *GA* Gestational age, *LBW* Low birth weight, *VLBW* Very low birth weight

LBW (≥ 1500 to < 2500 g), VLBW (≥ 1000 to < 1500 g)

Comparisons of pregnancy characteristics and outcomes between group A1 and A2 were shown in Supplementary Table S1 and 2. Of the 21 patients in group A2, 11 patients had a secondary MFPR of the co-twin, of whom 2 had a miscarriage, and the other 9 patients had one baby born. The other 10 patients had only one MFPR: the heartbeat of the co-twin had disappeared at the next visit 1 week later in 6 patients, and 5 had one baby born because miscarriage occurred in one patient within 1 month; in the remaining 4 patients, normal co-twin heartbeat and growth were observed on subsequent prenatal visits. One of these 4 patients underwent termination of pregnancy at 22^+5^ GA due to preterm premature rupture of membranes and intrauterine infection, and 3 patients gave birth to 2 babies each, all of whom survived. We were able to follow up with 2 patients via phone calls, and found that in both cases, the babies had normal development and prenatal MRI.

**Fig. 2:**
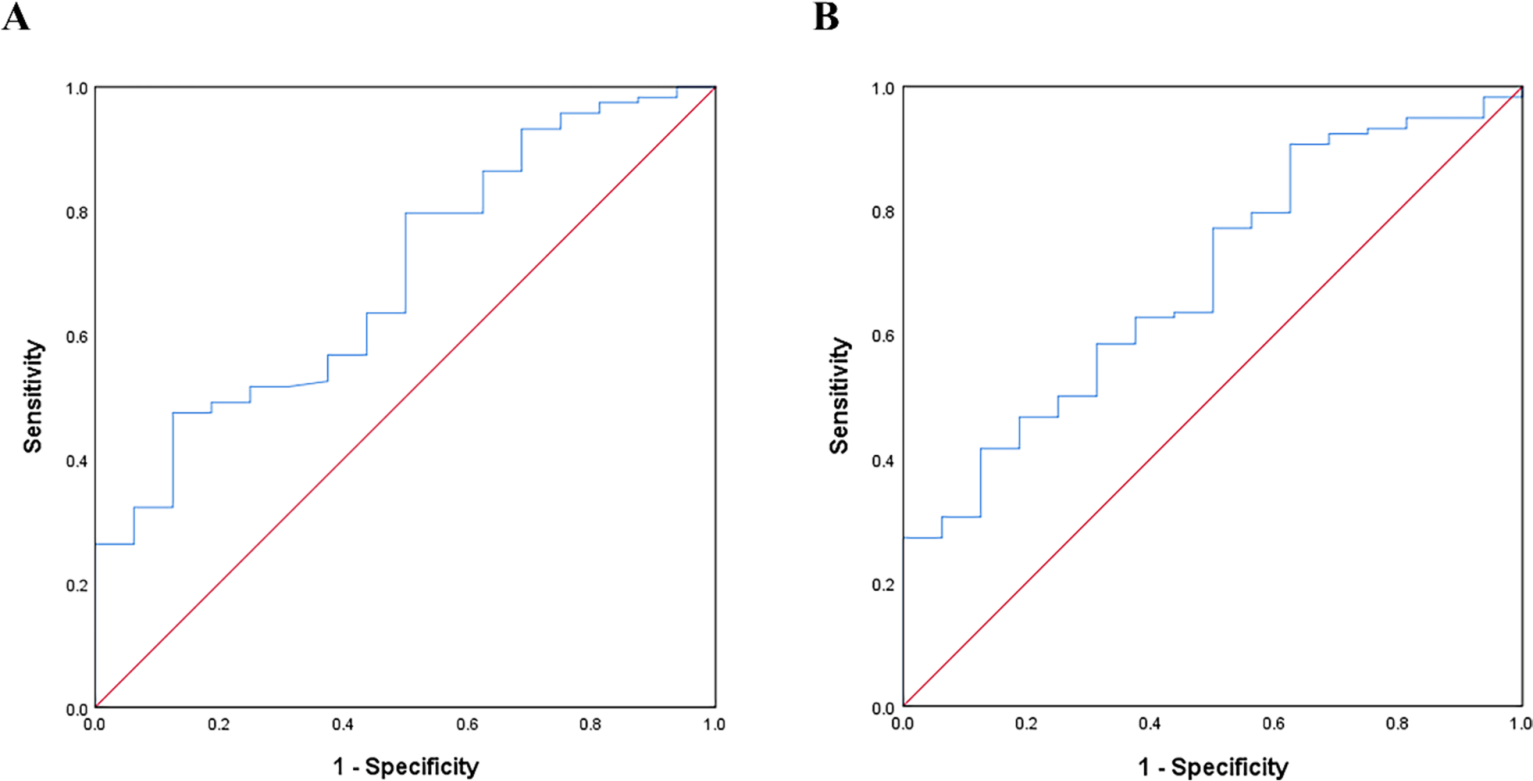
ROC curves. ROC curve of the adjusted model of NT difference (**A**) and the adjusted model of NT discordance (**B**) for predicting the co-twin death within 1 day after potassium chloride injection into one of the MC twins. ROC curve, receiver operating characteristic curve; NT, nuchal translucency; MC, monochorionic

To further explore factors that were potentially associated with the death of the MC co-twin after MFPR, we first compared the number of fetal punctures and dosage of potassium chloride during the MFPR procedure between groups A1 and A2. We found no between-group differences in these factors (Table 3). After excluding patients without data on ultrasound measurements at 11–13^+6^ weeks (group A1, 14 patients; group A2, 5 patients), we compared the patients with co-twin death within 1 day (*n* = 118) and patients with co-twin survival over 1 day (*n* = 16) by using logistic regression analysis (Table 4). On univariate analysis, no variable showed a significant association with MC co-twin death within 1 day. However, after adjustments for maternal age, parity, mode of conception, indication for MFPR, and GA at the time of MFPR, we found that a larger intertwin NT difference (OR, 9.27; 95% CI, 1.32–65.18; *P* = 0.025) and a greater NT discordance (OR, 1.04; 95% CI, 1.01–1.08; *P* = 0.045) were associated with MC co-twin death within 1 day. For adjusted models, ROC curves were plotted and compared (Fig. 2). The AUC was 0.694 (95% CI, 0.562–0.825) for the adjusted model of NT difference and 0.689 (95% CI, 0.558–0.819) for the adjusted model of NT discordance, with no significant differences between two models (*P* = 0.825). We also compared the intertwin NT difference and discordance between groups A2 and C, and found no significant disparity (Supplementary Table S3).

**Table 3:** Comparison of procedure-related characteristics between groups A1 and A2

**Characteristic**	**Group A1 (*n* = 132)**	**Group A2 (*n* = 21)**
Number of fetal punctures		
1	128 (97.0)	21 (100.0)
2	4 (3.0)	0
Dosage of potassium chloride (mL)	2.0 (2.0–3.0)	2.5 (2.0–5.5)

Data are expressed as median (interquartile range) or n (%)

The number of punctures required for secondary reduction in Group A2 are not included

**Table 4:** Logistic regression analysis of ultrasound measurements at 11–13^+6^ weeks

**Variable**	**Group A1**^**a**^ **(*n* = 118)**	**Group A2**^**a**^ **(*n* = 16)**	***P*** **value**	**Adjusted *P*** **value**^†^	**Odds ratio (95% CI)**
CRL difference (mm)	3.0 (1.7–6.0)	3.0 (1.0–6.0)	0.881	0.870	-
CRL discordance (%)	5.2 (2.8–10.3)	5.4 (1.3–10.4)	0.987	0.986	-
CRL discordance > 10%	30 (25.4)	4 (25.0)	0.971	0.979	-
NT difference (mm)	0.3 (0.2–0.7)	0.2 (0.1–0.5)	0.165	0.025	9.27 (1.32–65.18)
NT discordance (%)	22.6 (12.5–42.8)	15.6 (9.3–28.0)	0.135	0.045	1.04 (1.01–1.08)
Both NT < p95	104 (88.1)	14 (87.5)	-	-	-
One or both NT ≥ p95	9 (7.6)	2 (12.5)	0.999	0.999	-
One or both NT ≥ p99	5 (4.2)	0	0.832	0.946	-
AFV difference (mm)	4.0 (1.0–8.0)	4.0 (1.0–6.7)	0.384	0.498	-
AFV discordance (%)	14.0 (4.3–26.0)	15.3 (2.8–19.3)	0.472	0.633	-

Data are expressed as median (interquartile range) or n (%)

*AFV* Amniotic fluid volume, *CI* Confidence interval, *CRL* crown–rump length, *NT* Nuchal translucency thickness, *p95* 95^th^ percentile, *p99* 99^th^ percentile

^**a**^Excluding patients without data on ultrasound measurements at 11–13 ^+6^ weeks

^†^Adjusted for maternal age, parity, mode of conception, indication for fetal reduction, and gestation age at the time of fetal reduction

## Discussion

The present study showed that in DC triplet pregnancies, reduction of the MC twins resulted in fewer preterm births than those observed among women who underwent reduction of the singleton fetus, and the outcomes were comparable among women with injection into either one or both MC twins. Furthermore, we found that large intertwin NT difference/discordance was significantly associated with co-twin death within 1 day after potassium chloride injection into one of the MC twins.

A systematic review has reported similar results in that the rate of early preterm delivery after the reduction of the singleton fetus in DC triplets was 17.6% (95% CI, 6.2–41.0%) [9]. Studies have shown that both preterm birth and LBW are important factors affecting the short-term and long-term outcomes of infants [19–21]. Moreover, women who decide to reduce the singleton fetus would continue with the MC twin pregnancies, which may have unique complications such as TTTS and TAPS [22, 23]. In the present study, no such complications nor any miscarriages occurred after the reduction of the singleton fetus, probably because of the small sample size of this group (*n* = 12) and careful risk assessment of TTTS before reduction. Therefore, from the perspective of lowering the risk of preterm birth and potential complications, reduction of the MC twin pair is a better choice for women with DC triplets.

In our study, the rates of pregnancy loss were similar between women with potassium chloride injection into either one or both MC twins. Our study results suggest that the procedural effects, such as the number of fetal punctures and dosage of potassium chloride, on the preserved fetus may be negligible, which is a topic that has been little studied before and may need to be confirmed in studies with larger sample size. As the clinical outcomes, including the pregnancy outcomes and complications, were all comparable between potassium chloride injection into one or both MC twins, we suppose that though potassium chloride injection into one of the MC twins is not a routine program, it can be an alternative MFPR program in selected cases depending on the assessment of the operation difficulty, convenience of follow-up, and the parents’ wishes.

To our knowledge, only 1 previous study has reported that 15.3% of the co-twins survived after potassium chloride injection into one of the MC twins, which is consistent with our result (21/153,13.7%) [13]. In the above study, it was supposed that the dosage of potassium chloride precisely caused the death of just one fetus, but no further analysis was performed. In our study, there was no difference in the number of fetal punctures or the dosage of potassium chloride between groups A1 and A2, suggesting that the procedure-related characteristics were not associated with co-twin death. However, we found that larger intertwin NT difference and discordance were significantly associated with co-twin death within 1 day.

As communicating vessels are abundant in the MC placenta [24], co-twin death is likely to occur because of the flow of potassium chloride through the placental vascular shunt or because of acute hemodynamic changes in the surviving twin due to blood transfusion to the injected fetus [12]. Studies have shown that intertwin NT discordance is positively associated with the risk of TTTS and early fetal death, and researchers have proposed that this could be a sign of asymmetrical flow in the first trimester [25, 26]. Considering the above findings and our results, we supposed that large intertwin NT difference and discordance independent of fetal abnormality indicate more intertwin communicating vessels in the placenta, so when one of the MC twins is reduced, the co-twin is more susceptible and has a higher risk of subsequent death. The intertwin NT difference was similar in groups C and A2, which might partly explain why there was no TTTS in group C, and might be a potential risk assessment before reduction.

Although co-twin death has been reported in 15% of cases in MC twins complicated with sIUFD [27], few studies include sIUFD in the first trimester, and the lowest GA at which sIUFD may cause damage to the co-twin is unknown. One small study with 9 MC twin pregnancies in which sIUFD occurred as a complication between the 5^th^ and 11^th^ gestational week (mean, 7.4 weeks) reported poor prognoses for the co-twin [28]. There have also been case reports of severe neurological injury of the co-twin after sIUFD in the first trimester in an MC twin pregnancy [29] and an MC triplet pregnancy [30]. In our study, potassium chloride injection into one of the MC twins, a situation similar to sIUFD, was also performed mainly in early pregnancy, and co-twin death occurred within 1 week in most cases, suggesting that there may be abundant communicating vessels in early MC twin pregnancies. This indicates that the prognosis of sIUFD in early pregnancies may be related to the abundance of placental vascular shunt, but more studies and evidence are needed.

The strength of our study is that we explored how potassium chloride injection into one of the MC twins affected the co-twin and the factors that potentially influenced these effects, which have been little studied thus far. Our findings may provide evidence for future research on the effects of early MC placental anastomosis in MC twins with complications. However, there are several limitations in the present study. One is the relatively small sample size, which might lead to large instability and variability of the results. For example, the wide 95% CI of intertwin NT difference and difference of birth weight between group A1/A2, and there was no miscarriage in group C. A last limitation is that this is a single-center retrospective study, which is affected by local medical resource availability and patient compliance, so the MFPR program of potassium chloride injection into one of the MC twins may not be suitable in other regions and countries. Further, the association between intertwin NT difference and vascular anastomoses needs to be validated using three-dimensional ultrasonography or other techniques [31].

## Conclusions

In conclusion, our study showed that in women with DC triplet pregnancies who underwent MFPR by potassium chloride injection, the outcomes were comparable among women with injection into either one or both MC twins. In approximately 90% of pregnancies, co-twin death occurred within 1 day after injection into one of the MC twins, and cases with larger intertwin NT difference/discordance were more vulnerable. These results are helpful for improving consultation before MFPR in DC triplet pregnancies, and suggest that intertwin NT difference/discordance might be an indication of early vascular anastomoses in the MC placenta.

### Supplementary Information

**Additional file 1:** **Table S1. **Comparison of pregnancy characteristics between group A1 and A2. **Table S2. **Obstetric outcomes comparison between group A1 and A2. **Table S3. **Comparison of NT between groups A2 and C.

## Data Availability

All data analyzed during this study are included in this published article and its supplementary information file. The datasets analyzed during the current study are available from the corresponding author on reasonable request.

## Abbreviations

MFPR: Multifetal pregnancy reduction
DC: Dichorionic
KCl: Potassium chloride
MC: Monochorionic
NT: Nuchal translucency
ART: Assisted reproductive technology
TTTS: Twin-to-twin transfusion syndrome
TAPS: Twin anemia-polycythemia sequence
sIUFD: Single intrauterine fetal death
CRL: Crown-rump length
AFV: Amniotic fluid volume
GA: Gestational age
LBW: Low birth weight
VLBW: Very low birth weight
OR: Odds ratio
ROC: Receiver operator characteristic
AUC: Areas under the curve
TOP: Termination of pregnancy
NND: Neonatal death
IQR: Interquartile range
