# Supplementary material for: Intertwin nuchal translucency difference predicts the chance of co-twin death after fetal reduction in dichorionic triplet pregnancies: a retrospective analysis study
Source: BMC Pregnancy Childbirth. 2023 Oct 23;23:747. doi: 10.1186/s12884-023-06064-9 (PMC10594729; doi:10.1186/s12884-023-06064-9)
Supplement: Supplementary file 1 — Additional file 1: Table S1. Comparison of pregnancy characteristics between group A1 and A2. Table S2. Obstetric outcomes comparison between group A1 and A2. Table S3. Comparison of NT between groups A2 and C. [file 12884_2023_6064_MOESM1_ESM.docx]

**Supplementary tables**

**Table S1.** Comparison of pregnancy characteristics between group A1 and A2

| **Characteristic** | **Group A1**  **(n = 132)** | **Group A2**  **(n = 21)** | ***P* value** |
| --- | --- | --- | --- |
| Age (years) | 31 (28–34) | 32 (30–34) | 0.803 |
| Parity |  |  | 0.962 |
| Nulliparous | 81 (61.4) | 13 (61.9) |  |
| Parous | 51 (38.6) | 8 (38.1) |  |
| Mode of conception |  |  | 0.990 |
| Spontaneous | 19 (14.4) | 3 (14.3) |  |
| ART | 113 (85.6) | 18 (85.7) |  |
| Indication for MFPR |  |  | 0.425 |
| Triplets | 110 (83.3) | 16 (76.2) |  |
| Fetal anomaly | 22 (16.7) | 5 (23.8) |  |
| GA at MFPR (weeks) | $\text{12}^{\frac{\text{5}}{\text{7}}}$($\text{12}^{\frac{\text{1}}{\text{7}}}$–$\text{13}^{\frac{\text{4}}{\text{7}}}$) | 13 ($\text{12}^{\frac{\text{2}}{\text{7}}}$–$\text{15}^{\frac{\text{4}}{\text{7}}}$) | 0.134 |

Data are expressed as median (interquartile range) or n (%).

ART, assisted reproduction technology; MFPR, multifetal pregnancy reduction; GA, gestational age

| **Outcome** | **Group A1**  **(n = 132)** | **Group A2**  **(n = 21)** | ***P* value** |
| --- | --- | --- | --- |
| Pregnancy loss |  | | 0.210 |
| Miscarriage | 6 (4.5) | 3 (14.3) |  |
| TOP | 6 (4.5) | 1 (4.8) |  |
| Live birth |  |  | <0.001 |
| 1 | 120 (90.9) | 14 (66.7) | <0.05 |
| 2 | 0 | 3 (14.3) | <0.05 |
| At least 1 | 120 (90.9) | 17 (81.0) | 0.213 |
| NND | 1 (0.8) | 0 | 0.706 |
| Delivery |  |  |  |
| GA at delivery (weeks) | 39.0 (38.0–40.0) | 38.3 (36.5–39.4) | 0.176 |
| 28–33^+6^ weeks | 9 (7.5) | 1 (5.9) | NS |
| 34–36^+6^ weeks | 13 (10.8) | 4 (23.5) | NS |
| >37 weeks | 98 (81.7) | 12 (70.6) | NS |
| Caesarean section | 68 (56.7) | 9 (52.9) | 0.772 |
| Birth weight (g) | 3050 (2800–3362) | 2780 (2500–3013) | 0.002 |
| ≥2500 g | 101 (84.2) | 15 (88.2) | NS |
| LBW | 16 (13.3) | 2 (11.8) | NS |
| VLBW | 3 (2.5) | 0 | NS |

**Table S2.** Obstetric outcomes comparison between group A1 and A2

Data are expressed as median (interquartile range) or n (%).

NS, non-significant (*P* <0.05); TOP, termination of pregnancy; NND, neonatal death; GA, gestational age; LBW, low birth weight; VLBW, very low birth weight

LBW (≥1500 to <2500 g), VLBW (≥1000 to <1500 g)

| **Characteristic** | **Group A2^*^**  **(n = 16)** | **Group C**  **(n = 13)** | ***P* value** | **Adjusted *P* value^†^** | **Odds ratio (95% CI)** |
| --- | --- | --- | --- | --- | --- |
| NT difference (mm) | 0.2 (0.1–0.5) | 0.3 (0.2–0.4) | 0.881 | 0.091 | - |
| NT discordance (%) | 15.6 (9.3–28.0) | 21.4 (12.3–31.5) | 0.749 | 0.099 | - |
| Both NT < p95 | 14 (87.5) | 13 (100) | - | - | - |
| One or both NT ≥ p95 | 2 (12.5) | 0 | 0.999 | 0.999 | - |
| One or both NT ≥ p99 | 0 | 0 | - | - | - |

**Table S3.** Comparison of NT between groups A2 and C

Data are expressed as median (interquartile range) or n (%).

**^*^**Excluding patients without data on ultrasound measurements at 11–13^+6^ weeks

^†^Adjusted for maternal age, parity, mode of conception, indication for fetal reduction, and gestational age at the time of fetal reduction

AFV, amniotic fluid volume; CI, confidence interval; CRL, crown–rump length; NT, nuchal translucency thickness; p95, 95^th^ percentile; p99, 99^th^ percentile
